# Role of putative voltage-sensor countercharge D4 in regulating gating properties of Ca_V_1.2 and Ca_V_1.3 calcium channels

**DOI:** 10.1080/19336950.2018.1482183

**Published:** 2018-09-26

**Authors:** Pierre Costé de Bagneaux, Marta Campiglio, Bruno Benedetti, Petronel Tuluc, Bernhard E. Flucher

**Affiliations:** a Department of Physiology and Medical Physics, Medical University of Innsbruck, Innsbruck, Austria; b Institute of Experimental Neuroregeneration Spinal Cord Injury and Tissue Regeneration Center Salzburg (SCI-TReCS), Paracelsus Medical University, Salzburg, Austria; c Department of Pharmacology and Toxicology, University of Innsbruck, Innsbruck, Austria

**Keywords:** Alternative splicing, Voltage gated calcium channels, voltage-sensing

## Abstract

Voltage-dependent calcium channels (Ca_V_) activate over a wide range of membrane potentials, and the voltage-dependence of activation of specific channel isoforms is exquisitely tuned to their diverse functions in excitable cells. Alternative splicing further adds to the stunning diversity of gating properties. For example, developmentally regulated insertion of an alternatively spliced exon 29 in the fourth voltage-sensing domain (VSD IV) of Ca_V_1.1 right-shifts voltage-dependence of activation by 30 mV and decreases the current amplitude several-fold. Previously we demonstrated that this regulation of gating properties depends on interactions between positive gating charges (R1, R2) and a negative countercharge (D4) in VSD IV of Ca_V_1.1. Here we investigated whether this molecular mechanism plays a similar role in the VSD IV of Ca_V_1.3 and in VSDs II and IV of Ca_V_1.2 by introducing charge-neutralizing mutations (D4N or E4Q) in the corresponding positions of Ca_V_1.3 and in two splice variants of Ca_V_1.2. In both channels the D4N (VSD IV) mutation resulted in a  ̴5 mV right-shift of the voltage-dependence of activation and in a reduction of current density to about half of that in controls. However in Ca_V_1.2 the effects were independent of alternative splicing, indicating that the two modulatory processes operate by distinct mechanisms. Together with our previous findings these results suggest that molecular interactions engaging D4 in VSD IV contribute to voltage-sensing in all examined Ca_V_1 channels, however its striking role in regulating the gating properties by alternative splicing appears to be a unique property of the skeletal muscle Ca_V_1.1 channel.

## Introduction

The pore-forming α_1_ subunits of voltage-gated calcium channels (Ca_V_) are composed of four homologous but non-identical domains (repeats I, II, III, IV), each consisting of six membrane-spanning helices (S1-S6). Helices S1 through S4 of each repeat form voltage-sensing domains (VSD); helices S5, S6 and the connecting P-loop of all four repeats together form the channel pore [1,2]. Membrane depolarization moves the positively charged S4 helices outward, causing conformational change in the VSDs that activates channel opening. The energetically unfavorable transition of the positive S4 gating charges across the plane of the membrane is facilitated by sequential exchange of ion pair partners with negatively charged residues (countercharges) in the other helices of the VSDs [3–6]. Particularly an inner negative cluster functions as charge transfer center through which the S4 charges (R1, R2, R3, K4) transit upon activation and deactivation [7].

**Figure 1.**
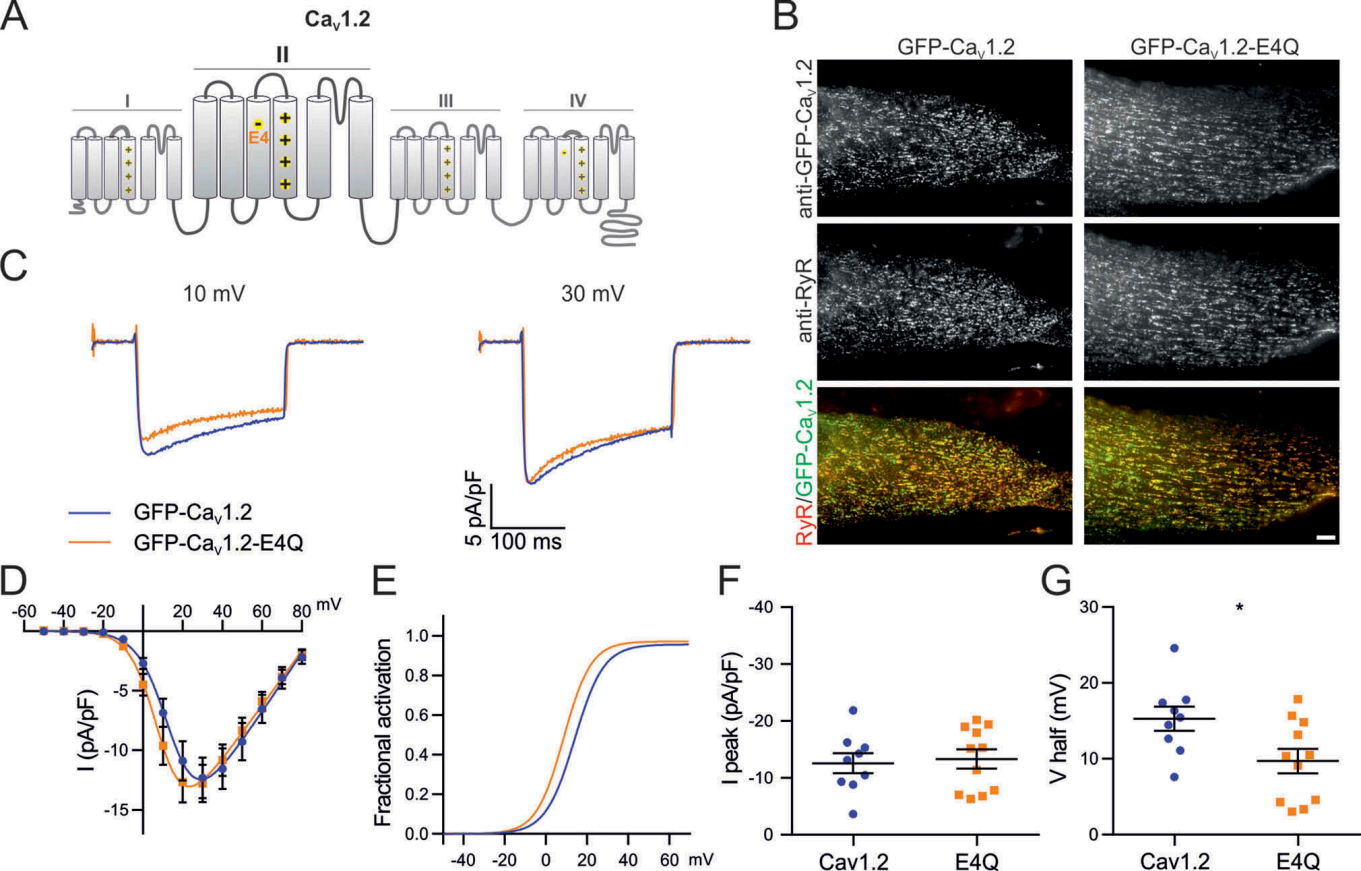
(A) Protein sequence alignment of human high voltage-activated calcium channels IVS3, IVS3-S4 loop, IVS4. The negative charge D4 is conserved as well as its putative interaction partners R1 and R2. The IVS3-S4 loops of all L-type channels (Ca_V_1) are subject to alternative splicing. The sequence of the rabbit IIS3, IIS3-S4 loop and IIS4 is indicated below to show common features. (B) Working model representing the mechanism by which alternative splicing of the exon 29 modulates the biophysical properties of Ca_V_1.1 (modified from [11]). In the intermediate state, D4 forms an interaction with R1 only in Ca_V_1.1e, facilitating the transition to the activated state, and therefore resulting in an improved voltage sensitivity of activation compared to that of Ca_V_1.1a. In the activated state, D4 of Ca_V_1.1e interacts with both R1 and R2, stabilizing the activated state and increasing the opening probability, whereas in Ca_V_1.1a, the inclusion of exon 29 decreases the number and strength of hydrogen bonds between D4, R1 and R2. (C) Fractional activation curves of Ca_V_1.1, Ca_V_1.2, Ca_V_1.3, and Ca_V_2.1 channels recorded in myotubes or HEK cells show the broad range of the voltage-dependence of activation of different voltage gated calcium channels.

Recently our laboratory identified an additional countercharge (D1196, short D4) in the outer segment of the S3 helix of the fourth VSD that is essential for the function and modulation of Ca_V_1.1 gating [8]. Rosetta homology modeling and site-directed mutagenesis suggested a model according to which in the activated state the negatively charged D4 establishes hydrogen bonds with the two outermost arginines (R1 and R2) of the IVS4 helix (Figure 1(B)). When D4 or either one of the putative interaction partners (R1, R2) were mutated in Ca_V_1.1e, the voltage-dependence of activation was shifted by  ̴20 mV towards more positive potentials and the current amplitude was dramatically reduced. Interestingly, this mirrored the effect of insertion of exon 29 into the extracellular loop connecting helices IVS3 and IVS4 (IVS3-S4 linker) that causes a similar 30 mV shift in voltage-dependence of activation and reduction of current density [9,10]. Therefore, this molecular interaction within VSD IV of Ca_V_1.1 facilitates channel gating and provides a means for regulation of the gating properties by alternative splicing. Consistent with this model, structure modeling indicated that insertion of exon 29 reduces the number and strength of the hydrogen bonds between D4 and R1/R2, thus perturbing both, the entry into the activated state, and/or the stabilization of the activated state [8].

Voltage-dependent calcium channels activate over a wide range of membrane potentials, tuned to function in many different physiological processes [12]. Ca_V_1.1a is the calcium channel least responsive to depolarization. Its voltage-dependence beyond the physiological range (Figure 1(C)) enables it to activate EC coupling without simultaneous calcium influx [13]. Ca_V_1.3 is the most responsive L-type calcium channel, ideally suited to function as pacemaker in neurons and the sinoatrial node [14]. Ca_V_1.2 – the predominant L-type calcium channel in the cardio-vascular system and in the brain – has an intermediate voltage-dependence of activation [1]. Interestingly, the negatively charged amino acid D4 in IVS3 is highly conserved throughout the Ca_V_ family and all L-, PQ-, and N-type channels comprise functionally important alternatively spliced exons in the IVS3-S4 linker (Figure 1(A)) [9,15–18]. Therefore we hypothesized that the D4-R1/R2 interaction mechanism, previously identified in Ca_V_1.1, may be involved in adjusting the specific voltage-dependences of activation and its regulation by alternative splicing also in other Ca_V_1 channels.

The four VSDs of Ca_V_ channels are structurally homologous but not identical [2,19]. Our recent mutagenesis studies unambiguously identified VSD IV as the major determinant of the voltage-dependence of Ca_V_1.1, whereas VSD I determines the kinetics of activation [8,10]. In contrast, recent voltage-clamp fluorometry studies demonstrated that in Ca_V_1.2 voltage-dependence of activation primarily is determined by VSDs II and III, whereas VSD IV does not contribute to it at all [20]. These conflicting findings indicate different roles of the four VSDs in Ca_V_1.1 and Ca_V_1.2 channels. Interestingly, VSD II (but not VSDs I and III) contains a conserved negatively charged residue (E510, short E4) in the position corresponding to D4 of VSD IV (Figure 1(A)). Thus, it is possible that molecular interactions similar to the D4-R1/R2 interactions in VSD IV are in place in VSD II. There they might participate in determining the specific voltage-sensitivity of Ca_V_1 channels, but probably not to their modulation, because the S3-S4 linker of the second VSD does not contain alternatively spliced exons.

Here we applied site directed mutagenesis and patch-clamp electrophysiological analysis to address the question as to whether the molecular mechanism (D4-R1/R2 interaction) in VSD IV of Ca_V_1.1 represents an isoform-specific feature of Ca_V_1.1 or a general mechanism for determining the gating properties of several Ca_V_ channels. Our data demonstrate that in Ca_V_1.2 and Ca_V_1.3 the countercharge D4 is involved in regulating the voltage-dependence of activation and/or the current density, although with a narrower dynamic range compared to Ca_V_1.1. Thus, the same mechanism that determines the specific voltage-dependence and amplitude of calcium currents in Ca_V_1.1 is utilized to fine tune the gating properties of Ca_V_1.2 and Ca_V_1.3.

## Results

### Role of the putative D4-R1/R2 interaction in regulating the gating properties of Ca_v_1.3

Ca_V_1.3 is the member of the Ca_V_1 channel family with the most left-shifted voltage sensitivity (Figure 1(C)). Moreover, a recent study demonstrated that its voltage-dependence of activation is differentially regulated by alternative splicing of exon 32 in the IVS3-S4 linker [16]. Therefore this channel is well suited to test the hypothesis that – in analogy to the D4-R1/R2 voltage-sensing interaction recently identified in Ca_V_1.1 [8,10] – D4 in the VSD IV is an important determinant of the gating properties of other L-type calcium channels as well. If in Ca_V_1.3 the negatively charged D4 (D1283) also acts as countercharge for the positive gating charges R1 and R2, and if this interaction is important for determining the sensitivity of Ca_V_1.3 to relatively low voltages, then charge neutralization of D4 is expected to cause a right-shift in the voltage-dependence of activation.

**Figure 2.**
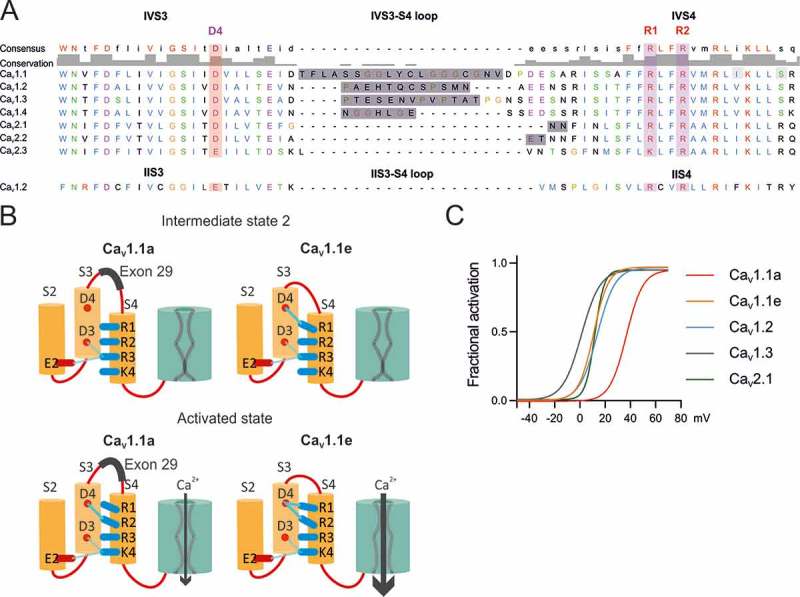
Charge-neutralization of D4 in VSD IV of Ca_V_1.3 affects voltage sensitivity and peak current density. (A) Domain structure of Ca_V_1.3 indicating the location of D4 in the outer S3 helix of VSD IV. (B) GFP-Ca_V_1.3 and mutant GFP-Ca_V_1.3-ΔE32-D4N are targeted to triads of dysgenic (Ca_V_1.1^−/-^) myotubes. Scale bar, 10 μm. (C) Representative calcium currents of GFP-Ca_V_1.3-ΔE32 (blue) and mutant GFP-Ca_V_1.3-ΔE32-D4N (orange) at 0 mV and + 20 mV. (D) I/V curves and (F) the scatter plot of the current density (I peak) show that D4N has a reduced current density. (E) Factional activation curves and (G) scatter plot of the V½ indicate that voltage sensitivity was reduced significantly by the charge neutralization of D4. (Mean± SEM; *p ≤ 0.05; **p ≤ 0.01; n = 9,10; Unpaired t-test).

To answer these questions we mutated the IVS4 D4 residue to an asparagine (D1283N) in a GFP-tagged Ca_V_1.3 construct lacking exon 32 (Figure 2(A)). Note, that in our previous work, inclusion of the analogous exon 29 in Ca_V_1.1a masked the effect of the D4N mutation [8]. Therefore, we performed the mutation in the splice variant lacking exon 32 to circumvent potential effects of exon inclusion on the putative D4-R1/R2 interaction. GFP-Ca_V_1.3-ΔE32 and GFP-Ca_V_1.3-ΔE32-D4N were expressed and analyzed in dysgenic myotubes. These Ca_V_1.1-null muscle cells are a well-established expression system for L-type calcium channels. Previously we have demonstrated that the L-type calcium channels Ca_V_1.2 and Ca_V_1.3 can be expressed in dysgenic myotubes, are correctly targeted into triads and/or peripheral junctions, express L-type calcium currents, and restore excitation-contraction coupling [21,22]. Figure 2(B) shows GFP-Ca_V_1.3-ΔE32 and the point mutant GFP-Ca_V_1.3-ΔE32-D4N expressed in dysgenic myotubes and immunofluorescence labeled with anti-GFP and anti-ryanodine receptor type 1 (anti-RyR1). Both, wildtype and mutant channels are equally expressed and distributed in clusters, representing triads and peripheral junctions as evidenced by the colocalization with RyR1.

**Figure 3.**
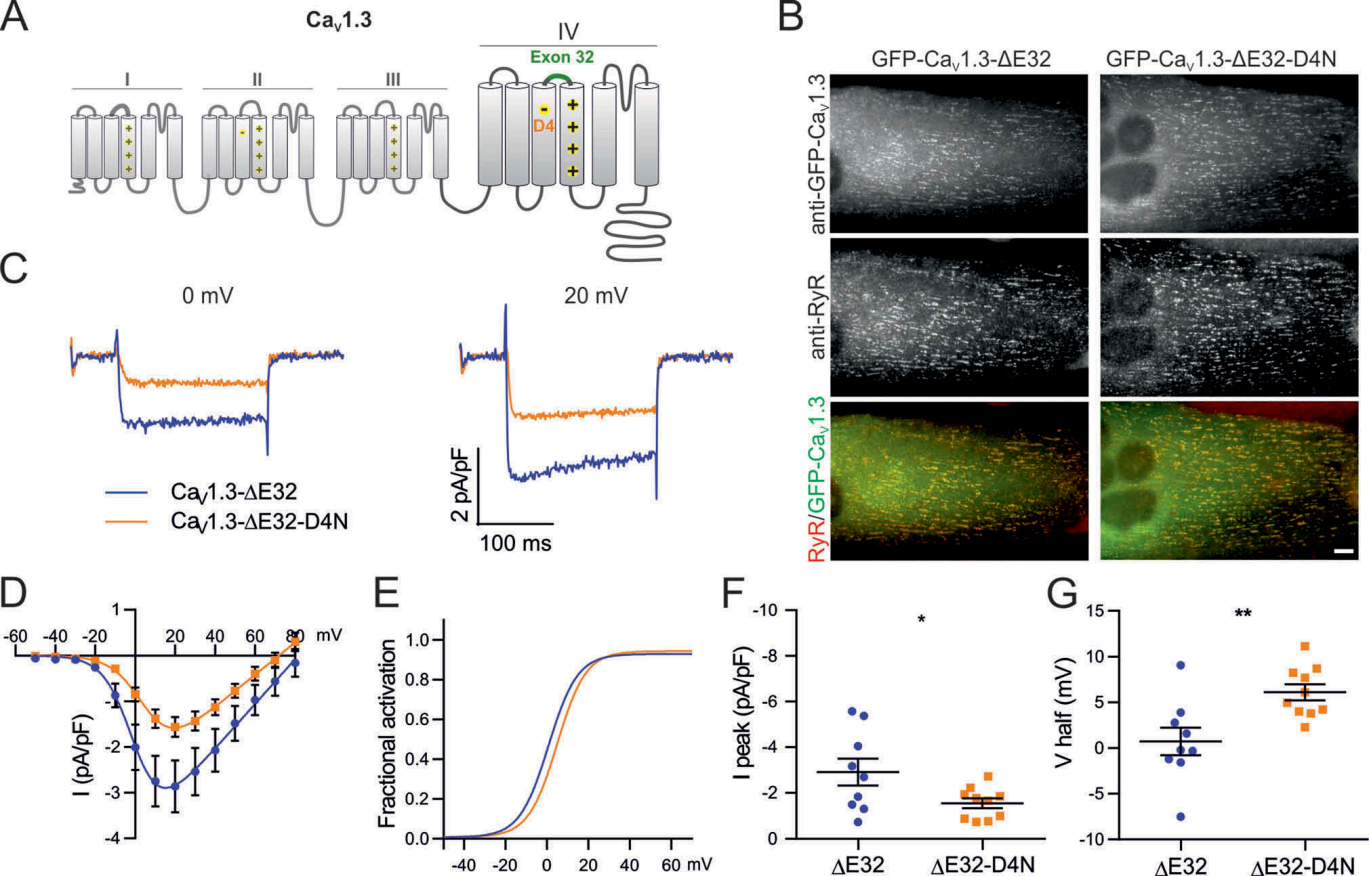
Expression and triad targeting of Ca_V_1.2 constructs. Dysgenic myotubes (Ca_V_1.1^-/-^) were reconstituted with GFP-Ca_V_1.2, GFP-Ca_V_1.2-D4N, GFP-Ca_V_1.2-ΔE33, or GFP-Ca_V_1.2-ΔE33-D4N and double immunofluorescence labeled with anti-GFP and anti-RyR. All channel constructs are equally expressed and colocalized in clusters with the RyR1, indicative of their correct targeting into t-tubule/SR or plasma membrane/SR junctions. Scale bar, 10 μm.

The GFP fluorescence aided the selection of transfected myotubes for whole-cell patch-clamp analysis. Calcium currents were recorded in series of 200 ms test pulses increasing from −50 to +80 mV in 10 mV increments. Figure 2(C–E) show representative current traces, the mean current-voltage relationships, and the fractional conductance curves, respectively, of GFP-Ca_V_1.3-∆E32 and GFP-Ca_V_1.3-ΔE32-D4N. Charge neutralization of D4 (D1283N) in GFP-Ca_V_1.3-∆E32-D4N resulted in a significant 5.4 mV shift of the mean voltage of half-maximal activation (V½) to more depolarizing potentials (p = 0.0056; Figure 2(D,E,G); Table 1), as well as in a significant reduction of the mean current density (p = 0.0368; Figure 2(D,F); Table 1), without altering kinetics of activation nor inactivation (Table 1). These effects indicate an involvement of D1283 in IVS3 in voltage-sensing and channel gating of Ca_V_1.3. The right-shifted V½ and reduced current density are qualitatively similar to the effects of the analogous mutation in Ca_V_1.1, although considerably smaller [8]. But as in Ca_V_1.1, the magnitude of the right-shift of V½ corresponds to the reported effects of inclusion of the alternatively spliced exons in the IVS3-S4 linker [16].

**Table 1.** Current properties of Ca_V_1.3-ΔE32 and Ca_V_1.3-ΔE32-D4N. Numerical values represent mean ± SEM. Statistical differences between mutants and controls were determined with unpaired Student’s t-test. Difference against control is represented as *p ≤ 0.05; **p ≤ 0.01.

	I_peak_ (pA/pF)	Significance (P value)	V_1/2_ (mV)	Significance (P value)	V_rev_ (mV)	k_act_ (mV)	G_max_ (nS/nF)	Tau (ms)	I_res200_/I_peak_	n
Ca_V_1.3-ΔE32	−2.9 ± 0.6		0.7 ± 1.5		80.7 ± 4.9	7.6 ± 0.8	50.3 ± 8.4	1.9 ± 0.3	0.77 ± 0.04	9
Ca_V_1.3-ΔE32-D4N	−1.6 ± 0.2	* (0.0368)	6.1 ± 0.9	** (0.0056)	74.6 ± 5.1	7.9 ± 0.4	35.5 ± 5.2	2.6 ± 0.3	0.75 ± 0.03	10

### Role of the putative VSD IV D4-R1/R2 interaction in the regulation of Ca_v_1.2 gating properties by alternative splicing

Ca_V_1.2 is the most widely expressed L-type calcium channel with a voltage-dependence of activation in between that of Ca_V_1.3 and Ca_V_1.1a. Alike these two channels also the gating properties of Ca_V_1.2 are modulated by alternative splicing in the IVS3-S4 linker [15]. Therefore we next examined the potential involvement of the D4-dependent mechanism in the modulation of voltage-dependence of activation in wildtype and mutant (D1327N) Ca_V_1.2 with and without exon 33. Normal expression and targeting of all used channel constructs in dysgenic myotubes was verified by double immunofluorescence labeling (Figure 3).

**Figure 4.**
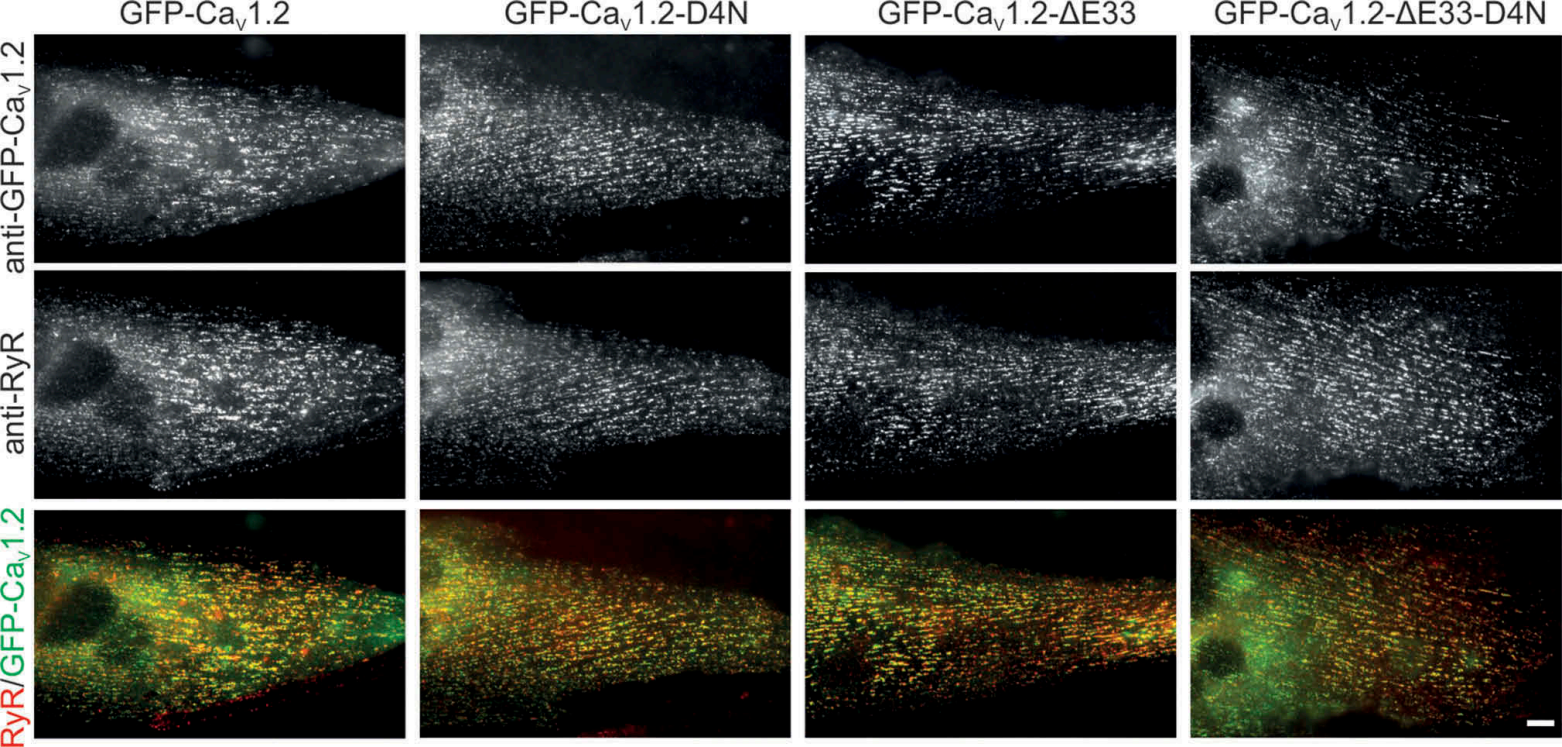
Effects of alternative splicing of exon 33 and of charge neutralization of E4 in VSD IV on the gating properties of Ca_V_1.2. (A) Domain structure of Ca_V_1.2 showing the location of the mutated D4 residue and the alternatively spliced exon 33 in VSD IV. (B,C) Representative calcium currents at + 10 mV and + 30 mV. (D) I/V curves and (F) scatter plots of the peak current densities show that charge neutralization of D4, but not exon 33 splicing, causes a significant reduction in current density. (E) The fractional activation curves and (G) scatter plots of the V½ show that inclusion of exon 33 and charge neutralization of D4 shift the voltage sensitivity of activation to more depolarized potentials. (Mean± SEM; *p ≤ 0.05; ***p ≤ 0.001; one-way ANOVA; n = 21,14,12,13).

Because alternative splicing of exon 33 typically is combined with other splicing events and the effects of its exclusion on gating properties depend very much on the specific combination of exons [23,24], we first examined the effect of excluding the exon 33 sequence from our rabbit GFP-Ca_V_1.2 construct (GFP-Ca_V_1.2-ΔE33) in the dysgenic myotube expression system. Consistent with published reports [15] exclusion of exon 33 caused a 6.3 mV (P = 0.052) shift of V½ to less depolarizing potentials without affecting peak current density (Figure 4(D–G); Table 2). Thus, alternative splicing of the Ca_V_1.2 IVS3-S4 linker shares a common effect on voltage-sensitivity with the analogous splicing events in Ca_V_1.1 and Ca_V_1.3, but unlike these channels it does not modify current density.

**Figure 5.**
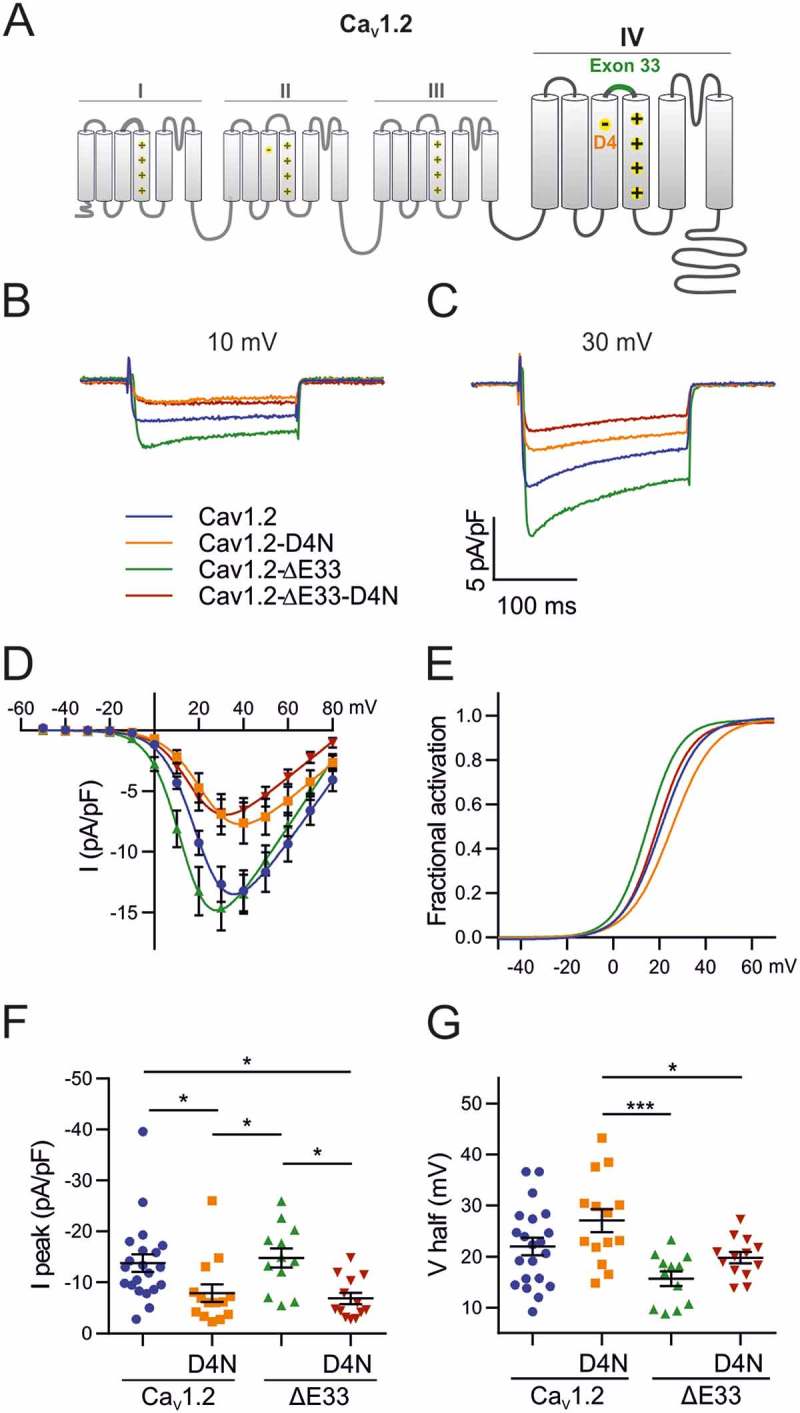
Charge neutralization of E4 in VSD II of Ca_V_1.2 affects the voltage sensitivity. (A) Domain structure of Ca_V_1.2 indicating the location of E4 in VSD II. (B) GFP-Ca_V_1.2 and mutant GFP-Ca_V_1.2-E4Q are equally expressed and targeted to triads of dysgenic myotubes. Scale bar, 10 μm. (C) Representative calcium currents at + 10 and + 30 mV depolarizations. (D) I/V curves (E) factional activation curves and the corresponding scatter plots of I peak (F) and V½ (G) show that the charge neutralization of E4 in VSD II shifts the voltage sensitivity to more hyperpolarized potentials. (Mean± SEM; *p ≤ 0.05; n = 9,11; Unpaired t-test).

To examine whether this modulation of GFP-Ca_V_1.2 gating properties by alternative splicing of exon 33 is effected by a mechanism involving the putative D4 countercharge, we next inserted the charge-neutralizing mutation D1327N into both GFP-Ca_V_1.2 and GFP-Ca_V_1.2-ΔE33 (Figure 4(A)). We hypothesized that if an interaction of the negatively charged D1327 with positive gating charges is necessary for modulation of V½ by alternative splicing of exon 33, then charge neutralization (D1327N) is expected to recapitulate the right-shift of V½ in GFP-Ca_V_1.2-ΔE33, but not in GFP-Ca_V_1.2 containing exon 33.

**Table 2.** Current properties of the different Ca_V_1.2 mutant and control constructs. Numerical values represent mean ± SEM. Statistical differences between mutants and controls were determined with one-way ANOVA (I_peak_: F_3, 56_ = 5.3; p = 0.0027; V_1/2_: F_3,56_ = 6.3; p = 0.0009), followed by a Holm-Sidak multiple comparison test. Differences against control are represented as *(P-value), difference against Ca_V_1.2-D4N are represented as †(P-value) and difference against Ca_V_1.2-ΔE33 are shown as ‡(P-value). Mean differences and their associated adjusted P-values for each of the six comparisons tested with the Holm-Sidak multiple comparison test.

	I_peak_ (pA/pF)	Significance (P value)	V_1/2_ (mV)		Significance (P value)	V_rev_ (mV)		k_act_ (mV)	G_max_ (nS/nF)		Tau (ms)	I_res200_/I_peak_	n
Ca_V_1.2	−13.8 ± 1.7		22.0 ± 1.7			93.48 ± 2.2		7.3 ± 0.4	275.5 ± 30.4		2.5 ± 0.29	0.62 ± 0.03	21
Ca_V_1.2-D4N	−7.9 ± 1.7	*(0.0393)	27.1 ± 2.3			95.5 ± 2.9		8.9 ± 0.4	169.2 ± 28.9		2.5 ± 0.18	0.60 ± 0.03	14
Ca_V_1.2-ΔE33	−14.9 ± 1.9	†(0.0393)	15.7 ± 1.4		†(0.0005)	88.25 ± 1.8		7.0 ± 0.4	282.8 ± 29.8		2.1 ± 0.31	0.61 ± 0.04	12
Ca_V_1.2-ΔE33-D4N	−6.9 ± 1.1	*(0.0233), ‡(0.0233)	19.8 ± 1.1		†(0.0379)	85.79 ± 2.1		7.7 ± 0.3	152.2 ± 21.2		2.6 ± 0.21	0.55 ± 0.04	13
		I_peak_					V_1/2_						
		Mean Difference		Adjusted P Value			Mean Difference			Adjusted P Value			
Ca_V_1.2 vs. Ca_V_1.2-D4N		−5.878		0.0393			−5.073			0.1032			
Ca_V_1.2 vs. Ca_V_1.2-ΔE33		1.025		0.8901			6.331			0.0515			
Ca_V_1.2 vs. Ca_V_1.2-ΔE33-D4N		−6.898		0.0233			2.199			0.3655			
Ca_V_1.2D4N vs. Ca_V_1.2ΔE33		6.903		0.0393			11.4			0.0005			
Ca_V_1.2D4N vs. Ca_V_1.2ΔE33-D4N		−1.02		0.8901			7.271			0.0379			
Ca_V_1.2ΔE33 vs. Ca_V_1.2ΔE33-D4N		−7.923		0.0233			−4.133			0.2539			

The data presented in Figure 4 show that the charge neutralizing mutation GFP-Ca_V_1.2-ΔE33-D4N significantly reduced peak current density to about half of control values (Table 2). The D4N mutation also right-shifted V½ by approximately 4 mV, but this shift remained below statistical significance (P = 0.103). These changes indicate an involvement of D4 in voltage sensing and channel gating. However, charge neutralization of D4 had very similar effects on GFP-Ca_V_1.2. Also in the presence of exon 33 the D1327N mutation caused a significant reduction of peak current density and an approximately 5 mV, although statistically insignificant, right-shift of V½ (Figure 4(D–G)). Conversely, alternative splicing of exon 33 significantly affected voltage-dependence of activation in GFP-Ca_V_1.2-D4N and showed a similar trend in GFP-Ca_V_1.2. This indicates that in Ca_V_1.2 channels, modulation of gating by alternative splicing of the IVS3-S4 linker or by charge neutralization of D4 act by independent mechanisms. Kinetics of activation or inactivation were not altered by charge neutralization or exon 33 exclusion (Table 2A).

### Is the analogous E4-R1/R2 interaction in VSD II of Ca_v_1.2 involved in the regulation of gating properties?

Although in both Ca_V_1.2 and Ca_V_1.3 neutralization of D4 in the fourth VSD affected voltage-dependence of activation and peak current density, the magnitude of these effects was substantially smaller than that previously observed in Ca_V_1.1 [8,10]. This could indicate that in Ca_V_1.2 and Ca_V_1.3 the putative D4-R1/R2 interaction is of minor importance for voltage-sensing, or it could indicate that the contribution of VSD IV to channel gating is smaller compared to Ca_V_1.1. In fact, a recent voltage-clamp fluorometry study indicated that in Ca_V_1.2 mainly VSDs II and III determine the gating properties, whereas the contribution of VSD IV is marginal [20]. Of the two presumably more relevant VSDs, only VSD II contains a conserved negatively charged residue (E632) in the position analogous to D4 in VSD IV (Figure 1(A)). Therefore we wondered whether in Ca_V_1.2 an interaction of the positive gating charges in IIS4 with E632 in IIS3 might be involved in the gating charge transition during voltage-sensing.

**Table 3.** Current properties of Ca_V_1.2 and Ca_V_1.2-E4Q. Numerical values represent mean ± SEM. Statistical differences between mutants and controls were determined with unpaired Student’s t-test. Difference against control is represented as *(P-value).

	I_peak_ (pA/pF)	Significance (P value)	V_1/2_ (mV)	Significance (P value)	V_rev_ (mV)	k_act_ (mV)	G_max_ (mV)	Tau (ms)	I_res200_/I_peak_	n
Ca_V_1.2	−12.6 ± 1.7		15.3 ± 1.6		86.7 ± 1.8	6.9 ± 0.4	233.1 ± 34.8	1.7 ± 0.2	0.55 ± 0.05	9
Ca_V_1.2-E4Q	−13.3 ± 1.7	n.s. (0.7620)	9.7 ± 1.6	* (0.0254)	87.5 ± 1.5	6.2 ± 0.5	305 ± 64.2	1.9 ± 0.2	0.52 ± 0.03	11

To test this possibility we generated a charge-neutralizing mutation (E632Q, short E4Q) in VSD II of GFP-Ca_V_1.2 (Figure 5(A)) and analyzed its current properties using patch-clamp recordings in dysgenic myotubes. If the negative charge of E632 is important for voltage sensing (e.g. acting as countercharges for R1 and R2 as in VSD IV of Ca_V_1.1), then its neutralization should right-shift the voltage-dependence of activation and/or diminish the peak current density. Figure 5(B) shows that the mutant channel GFP-Ca_V_1.2-E4Q is normally expressed and distributed in dysgenic myotubes. However, the electrophysiological analysis demonstrates that the current properties of GFP-Ca_V_1.2-E4Q were affected by the charge-neutralizing mutation (Figure 5(C–G)) differently to what was observed in the VSD IV D4N mutation. While peak current density of GFP-Ca_V_1.2-E4Q currents did not differ from that of the matched GFP-Ca_V_1.2 controls (Table 3), the V½ was significantly shifted toward more negative potential (p = 0.0254). This increase in voltage-sensitivity by the charge neutralizing mutation refutes the possibility that an intramolecular interaction involving E4 in VSD II facilitates the activation of Ca_V_1.2. If anything it would be consistent with a role of E4 in stabilizing a pre-open state.

## Discussion

Previously we identified a novel molecular mechanism that controls the voltage-dependence of current activation in the skeletal muscle isoform of voltage-gated calcium channels. It involves dynamic interactions of the outer gating charges (R1 and R2) with a newly identified countercharge (D4) in the S3 transmembrane segment of the fourth VSD. This interaction facilitates channel activation in the embryonic Ca_V_1.1e variant, but it is disrupted by alternative splicing in the extracellular IVS3-S4 linker of the adult Ca_V_1.1a splice variant, resulting in a striking right-shift of V½ and reduction of channel open probability. Whether this mechanism is unique to Ca_V_1.1 or plays a role in other L-type calcium channels remained to be elucidated.

The data presented here provide evidence for the involvement of this D4-R1/R2 interaction in VSD IV in the regulation of channel gating in other members of the L-type calcium channel family. In Ca_V_1.2 and in Ca_V_1.3 charge neutralization of D4 (D1327N, D1283N, respectively) caused a right-shift of V½ and a reduction of current density. Qualitatively these effects are consistent with the original observation in Ca_V_1.1 [8,10] and suggest that, similarly to the situation in the skeletal muscle channel isoform, D4 in Ca_V_1.2 and Ca_V_1.3 might be involved in voltage sensor function and facilitate channel activation by serving as countercharge for the outer gating charges R1 and R2 in VSD IV. Previous structure modeling and mutagenesis analysis in Ca_V_1.1 suggested that hydrogen bonds established between D4 and R1 during intermediate state 2 facilitate the transition into the activated state, resulting in the observed left-shifted V½. Additional hydrogen bonds subsequently established between D4 and R2 likely stabilize the activated state, thus resulting in an increased P_o_. Finding similar effects of D4N charge neutralization in Ca_V_1.2 and Ca_V_1.3 suggests that the principal function of D4 in VSD IV is a general feature of L-type calcium channels. Interestingly, in Ca_V_1.2 the effect of D4N charge neutralization on reducing the current amplitude was stronger than its effect on V½, suggesting that in this channel D4-R1/R2 interactions primarily function in stabilizing the activated state.

While qualitatively the effects of charge neutralization of D4 were similar in the three examined Ca_V_1 channels, the magnitude of the effects observed in Ca_V_1.2 and Ca_V_1.3 was much smaller than those previously found in the skeletal muscle isoform. Whereas in Ca_V_1.1 charge neutralization of D4 caused a  ̴20 mV right-shift of V½ and reduction of current density to 15% of control [8,9], in Ca_V_1.2 and Ca_V_1.3 current density was reduced to about half of control levels and the right-shift of V½ was only about 5 mV, and in Ca_V_1.2 did not reach statistical significance. Thus, it appears that this mechanism, although present, is of lesser importance for voltage sensing of VSD IV in Ca_V_1.2 and Ca_V_1.3. This does not exclude the possibility that the role of D4 as countercharge in the VSD IV of Ca_V_1.2 and Ca_V_1.3 might be essentially the same as in Ca_V_1.1, if the contribution of this VSD to channel gating were lower in Ca_V_1.2 and Ca_V_1.3. In that case, even perturbing an important mechanism of voltage sensing in VSD IV would only result in minor changes of the gating properties. This notion is consistent with recent findings indicating that in Ca_V_1.2 VSD IV contributes the least of all four VSDs to channel gating [20]. Another implication of this concept is that the specific contributions of individual VSDs differ between the Ca_V_1 family members. The limited effects of exon 32 insertion and D4N charge neutralization in Ca_V_1.3 suggest that also in this L-type calcium channel VSD contributes little to channel gating. Conversely, the big effects of exon 29 insertion and D4N charge neutralization in Ca_V_1.1 clearly demonstrate that in the skeletal muscle channel isoform the contribution of VSD IV to channel gating is substantially greater than in its close relatives Ca_V_1.2 and Ca_V_1.3. This explanation is corroborated by the different magnitudes of effects of alternative splicing in the IVS3-S4 linker which are also considerably smaller in Ca_V_1.2 [15] and Ca_V_1.3 [16], compared to Ca_V_1.1 [8,10].

Using voltage-clamp fluorometry Pantazis et al. [20] reported that in Ca_V_1.2 VSD II and VSD III together contribute about 85% of the energy necessary for channel opening, whereas the contribution of VSD IV is negligible. Because a negatively charged amino acid in the position corresponding to D4 in VSD IV is conserved in VSD II, neutralization of this charge in Ca_V_1.2 might affect voltage sensitivity in this channel similarly to neutralizing D4 in VSD IV of Ca_V_1.1. However, our data do not support this possibility, as charge neutralization of E632 in Ca_V_1.2 did not affect current density and even showed the opposite effect on voltage-dependence, causing a small but significant shift of the V_1/2_ to less depolarized potentials. Beyl and coworkers demonstrated that neutralization of the gating charges R1 and R2 in Ca_V_1.2 VSD II [25,26] resulted in little or no shifts of the voltage-dependence of activation. Together these findings dismiss the notion that interactions between R1/R2 and E4, acting as a counter charge, are important for VSD II function and constitute a major determinant of the specific gating properties of Ca_V_1.2.

Regarding the importance of D4 in IVS3 for modulation of gating properties by alternative splicing our data reveal differences between the members of the L-type calcium channel family. In Ca_V_1.3 the observed right-shift of V½ upon charge neutralization of D4 matched the voltage shift reported for insertion of exon 32 [16]. Thus, it is reasonable to assume that, as demonstrated for Ca_V_1.1, insertion of the alternatively spliced exon into the IVS3-S4 linker perturbs the interaction of D4 with R1 and R2 in Ca_V_1.3. However, in Ca_V_1.2 a similar effect of exon 33 insertion on V½ was observed with and without D4, and conversely, the effects of D4N charge neutralization on current density were observed with and without exon 33. Therefore, the effects of alternative exon 33 splicing on gating properties do not depend on the negative charge of D1327. Thus, Ca_V_1.1 and Ca_V_1.2 utilize distinct molecular mechanisms for modulation of current properties by alternative splicing in the IVS3-S4 linker. Whereas the interaction between D4 and R1/R2 in VSD IV appears to contribute to voltage-sensing of VSD IV in all three examined Ca_V_1 channels it is not the general mechanism for the regulation of gating properties in all L-type calcium channels. On a broader scale, the S3-S4 linker of the fourth VSD appears to be a hotspot for modulation of gating properties by alternative splicing and toxin binding in many voltage-gated cation channels [9,16–18,27]. This highlights the overall importance of this VSD for channel gating. However, the differential effects of alternative splicing in the IVS3-S4 linker on open probability, rate and voltage-dependence of activation in various Ca_V_1 and Ca_V_2 channels are consistent with the notion that multiple molecular mechanisms mediate these effects – the D4-R1/R2 interaction being one of them.

Together with our previous findings [8,10], our current results support the model that D4 acts as a counter charge for the voltage-sensing mechanism in the VSD IV of all examined Ca_V_1 channels. However, due to the different contributions of VSD IV to channel gating in L-type calcium channels, alternative splicing or experimental perturbations of this mechanism affect voltage-dependence of activation and current density far less in Ca_V_1.2 and Ca_V_1.3 than in Ca_V_1.1. Consequently, the D4-R1/R2 interaction in VSD IV is a major determinant of the characteristic gating properties of Ca_V_1.1, whereas in Ca_V_1.2 and Ca_V_1.3 it contributes to the fine tuning of channel gating. This differential importance of D4 in shaping current properties might on the one hand relate to the need of Ca_V_1.2 and Ca_V_1.3 to adjust their current properties to diverse cellular functions in the cardio-vascular system and the nervous system, and on the other hand to the unique dual function of Ca_V_1.1 as calcium channel in embryonic muscle and as voltage-sensor for EC coupling in mature skeletal muscle.

## Materials and methods

### Cloning procedures

All α_1_-subunits are N-terminally GFP-tagged and their expression is under the control of a CMV promoter. Sequence integrity of the all newly generated constructs was confirmed by sequencing (MWG Biotech, Martinsried, Germany). *GFP-Ca_V_1.3-ΔE33*: The human Ca_v_1.3α_1_-subunit (EU363339) comprising alternative exons 8a and 42, but not exon 33 (long C-terminal splice variant) was previously cloned into the pGFP^minus^ vector [28]. Briefly the SacII to SalI fragment of GFP-Ca_V_1.2 [29] containing the GFP-tag was inserted “in-frame” and upstream of the coding region of Ca_v_1.3, yielding GFP-Ca_V_1.3-ΔE33. *GFP-Ca_V_1.3-ΔE33-D4N*: The D1283N mutation neutralizes the fourth negative charge in the IVS3 of Ca_V_1.3 and was generated by SOE-PCR. Briefly, nt 3278–4842 of Ca_V_1.3 were PCR amplified with overlapping primers introducing the point mutation G > A at position nt 3847 in separate PCR reactions using GFP-Ca_V_1.3-ΔE33 as template. The two separate PCR products were then used as templates for a final PCR reaction with flanking primers to connect the nucleotide sequences. This fragment was then BglII/BstEII digested and cloned into the respective sites of GFP-Ca_V_1.3-ΔE33 yielding GFP-Ca_V_1.3-ΔE33-D4N. *GFP-Ca_V_1.2*: For the GFP-tagged rabbit Ca_V_1.2α_1_-subunit (X15539) cloning procedures were previously described [29]. *GFP-Ca_V_1.2-D4N:* The D1327N mutation neutralizes the fourth negative charge in the IVS3 of Ca_V_1.2 and was generated by SOE-PCR. Briefly, nt 3403–4728 of Ca_V_1.2 were PCR amplified with overlapping primers introducing the point mutation G > A at position nt 3979 in separate PCR reactions using GFP-Ca_V_1.2 as template. The two separate PCR products were then used as templates for a final PCR reaction with flanking primers to connect the nucleotide sequences. This fragment was then ApaI/BstEII digested and cloned into the respective sites of GFP-Ca_V_1.2 yielding GFP-Ca_V_1.2-D4N.

*GFP-Ca_V_1.2-ΔE33* and *GFP-Ca_V_1.2-ΔE33-D4N* (D1327N) lack exon 33 and were generated by SOE-PCR. Briefly, nt 3403–4728 of Ca_V_1.2 or Ca_V_1.2-D4N were PCR amplified with overlapping primers deleting exon 33 (aa 1335-PAEHTQCSPSM-1345) in separate PCR reactions using either GFP-Ca_V_1.2 or GFP-Ca_V_1.2-D4N as template. The two separate PCR products were then used as templates for a final PCR reaction with flanking primers to connect the nucleotide sequences. This fragment was then ApaI/BstEII digested and cloned into the respective sites of GFP-Ca_V_1.2 yielding GFP-Ca_V_1.2-ΔE33 and GFP-Ca_V_1.2-ΔE33-D4N. *GFP-Ca_V_1.2-E4Q:* The E632Q mutation neutralizes the third negative charge in the IIS3 of Ca_V_1.2 and was generated by SOE-PCR. Briefly, nt 1165–2766 of Ca_V_1.2 were PCR amplified with overlapping primers introducing the point mutation G > C at position nt 1893 in separate PCR reactions using GFP-Ca_V_1.2 as template. The two separate PCR products were then used as templates for a final PCR reaction with flanking primers to connect the nucleotide sequences. This fragment was then BamHI/AflII digested and cloned into the respective sites of GFP-Ca_V_1.2 yielding GFP-Ca_V_1.2-E4Q.

### Dysgenic myotubes cell culture and transfection

Myotubes from the dysgenic cell line GLT [30] were cultured as previously described. Briefly, cells were plated on 35 mm culture dishes and transfected with 0.5 μg of the desired Ca_V_1 subunit 4 days after plating using FuGENE HD transfection reagent (Promega). After 8 to 10 days in culture, transfected myotubes showing GFP fluorescence were analyzed by electrophysiology or fixed for immunolabeling. GLT myotubes endogenously express the auxiliary α_2_δ-1, β_1a_, and γ_1_ calcium channel subunits as well as STAC3 and the ryanodine receptor, enabling proper functional incorporation of the channel constructs in the triad junction [31–33].

### Immunofluorescence and antibodies

Paraformaldehyde-fixed cultures were immunolabeled as previously described [34] with rabbit polyclonal anti-GFP (1:10,000; Molecular Probes, Eugene, OR) and mouse monoclonal anti-RyR (34-C; 1:1000; Alexis Biochemicals, Lausanne, Switzerland) and fluorescently labeled with goat anti-rabbit Alexa-488 and secondary goat anti-mouse Alexa-594 (1:4000; Molecular Probes), respectively. Thus, the anti-GFP label and the intrinsic GFP signal were both recorded in the green channel. Samples were observed using a 63X, 1.4 NA objective Axioimager microscope (Carl Zeiss Inc., Oberkochen, Germany) and 14-bit images were captured with a cooled charge-coupled device camera (SPOT; Diagnostic Instruments, Stirling Heights, MI, USA) and Metaview image-processing software (Universal Imaging, West Chester, PA, USA). Image composites were arranged in Adobe Photoshop CS3 (Adobe Systems INC., San Francisco, CA, USA) and linear adjustments were performed to correct black level and contrast.

### Electrophysiology and data analysis

Calcium currents were recorded using the whole cell patch clamp technique in voltage clamp mode using an Axopatch 200B amplifier (Axon Instruments). Patch pipettes were pulled (Sutter instrument model P-1000) from borosilicate glass (Warner Instruments) and were filled with (mM) 145 Cs-aspartate, 2 MgCl2, 10 HEP ES, 0.1 Cs-EGTA, and 2 Mg-ATP (pH 7.4 with CsOH) and had a resistance between 1.5 and 3 MΩ. For the recording of the calcium currents, the myotubes were bathed in solution containing (mM) 10 CaCl2, 145 tetraethylammonium chloride, and 10 HEPES (pH 7.4 with tetraethylammonium hydroxide). Data acquisition and command potentials were controlled by Clampex software (version 10.6; Axon Instruments); analysis was performed using Clampfit 10.5 (Axon Instruments) and Sigma-Plot 8.0 (SPSS Science) software. The current-voltage dependence was fitted according to: *I *= *G*_max_ ⋅ (*V* − *V_rev_*)/1(1 + exp(−(*V* − *V*_1/2_)/*k*)), where G_max_ is the maximum conductance of the L-type calcium currents, V_rev_ is the extrapolated reversal potential of the calcium current, V_1/2_ is the potential for half maximal conductance, and k is the slope. The conductance was calculated using *G *= (−*I* ∗ 1000)/(*V* − *V_rev_*), and its voltage dependence was fitted according to a Boltzmann distribution: *G *= *G*_max_/(1 + exp(−(*V* − *V*_1/2_)/*k*)). The Kinetics of activation was best described by a mono-exponential function and its time constant (Tau) was used to compare the kinetics of each constructs, while their inactivation properties were determined as the residual current at 200ms (I_res_200). The statistical significance was calculated using t-test or one-way ANOVA as indicated in the legends of the tables.

## References

[1] Catterall WA. Voltage-gated calcium channels. Cold Spring Harb Perspect Biol. 2011;3:a003947. doi:10.1101/cshperspect.a003947. PMC3140680 21746798

[2] Wu J, Yan Z, Li Z, et al Structure of the voltage-gated calcium channel Cav1.1 complex. Science. 2015;350:aad2395. doi:10.1126/science.aad2395. 26680202

[3] Yarov-Yarovoy V, DeCaen PG, Westenbroek RE, et al Structural basis for gating charge movement in the voltage sensor of a sodium channel. Proc Natl Acad Sci U S A. 2012;109:E93-102. doi:10.1073/pnas.1118434109. PMC3258622 22160714

[4] Ahern CA, Payandeh J, Bosmans F, et al The hitchhiker’s guide to the voltage-gated sodium channel galaxy. J Gen Physiol. 2016;147:1–24. doi:10.1085/jgp.201511492. PMC4692491 26712848

[5] Lacroix JJ, Hyde HC, Campos FV, et al Moving gating charges through the gating pore in a Kv channel voltage sensor. Proc Natl Acad Sci USA. 2014;111:E1950–9. doi:10.1073/pnas. PMC4024920 24782544

[6] Catterall WA. Ion channel voltage sensors: structure, function, and pathophysiology. Neuron. 2010;67:915–928. doi:10.1016/j. PMC2950829 20869590

[7] Tao X, Lee A, Limapichat W, et al A gating charge transfer center in voltage sensors. Science. 2010;328:67–73. doi:10.1126/science. PMC2869078 20360102

[8] Tuluc P, Yarov-Yarovoy V, Benedetti B, et al Molecular interactions in the voltage sensor controlling gating properties of CaV calcium channels. Structure. 2016;24:261–271. doi:10.1016/j.str.2015.11.011. PMC7360434 26749449

[9] Tuluc P, Molenda N, Schlick B, et al A Cav1.1 Ca2+ channel splice variant with high conductance and voltage-sensitivity alters EC coupling in developing skeletal muscle. Biophys J. 2009;96:35–44. doi:10.1016/j.bpj.2008.09.027. PMC2710043 19134469

[10] Tuluc P, Benedetti B, Coste De Bagneaux P, et al Two distinct voltage-sensing domains control voltage sensitivity and kinetics of current activation in Ca V 1.1 calcium channels. J Gen Physiol. 2016;147:437–449. doi:10.1085/jgp.201611568. PMC4886277 27185857

[11] Flucher BE, Tuluc P How and why are calcium currents curtailed in the skeletal muscle voltage-gated calcium channels? J Physiol. 2017;595:1451–1463. doi:10.1113/JP273423. PMC5330888 27896815

[12] Lipscombe D, Andrade A, Allen SE Alternative splicing: functional diversity among voltage-gated calcium channels and behavioral consequences. Biochim Biophys Acta. 2013;1828:1522–1529. doi:10.1016/j.bbamem.2012.09.018. PMC3625486 23022282

[13] Melzer W, Herrmann-Frank A, Lüttgau H-C The role of Ca 2++ ions in excitation-contraction coupling of skeletal muscle fibres. Biochim Biophys Acta. 1995;1241:59–116. doi:10.1016/0304-4157(94)00014-5. 7742348

[14] Pinggera A, Striessnig J Cav 1.3 (CACNA1D) L-type Ca(2+) channel dysfunction in CNS disorders. J Physiol. 2016;594:5839–5849. doi:10.1113/JP270672. PMC4823145 26842699

[15] Tang ZZ, Liang MC, Lu S, et al Transcript scanning reveals novel and extensive splice variations in human L-type voltage-gated calcium channel, Cav1.2 α1 subunit. J Biol Chem. 2004;279:44335–44343. doi:10.1074/jbc.M407023200. 15299022

[16] Liu N, Liu Y, Yang Y, et al Linker flexibility of IVS3-S4 loops modulates voltage-dependent activation of L-type Ca 2+ channels. Channels (Austin). 2017;11:34–45. doi:10.1080/19336950.2016. PMC5279877 27362349

[17] Bourinet E, Soong TW, Sutton K, et al Splicing of alpha 1A subunit gene generates phenotypic variants of P- and Q-type calcium channels. Nat Neurosci. 1999 May;2(5):407–15. doi:10.1038/8070. 10321243

[18] Lin Z, Lin Y, Schorge S, et al Alternative splicing of a short cassette exon in alpha1B generates functionally distinct N-type calcium channels in central and peripheral neurons. J Neurosci. 1999;19:5322–5331. doi:10.1523/JNEUROSCI.19-13-05322.1999. PMC6782300 10377343

[19] Wu J, Yan Z, Li Z, et al Structure of the voltage-gated calcium channel Cav1.1 complex. Nature. 2016;537:191–196. doi:10.1038/nature19321. 27580036

[20] Pantazis A, Savalli N, Sigg D, et al Functional heterogeneity of the four voltage sensors of a human L-type calcium channel. Proc Natl Acad Sci USA. 2014;111:18381–18386. doi:10.1073/pnas.1411127112. PMC4280600 25489110

[21] Kasielke N, Obermair GJ, Kugler G, et al Cardiac-type EC-coupling in dysgenic myotubes restored with Ca2+ channel subunit isoforms alpha1C and alpha1D does not correlate with current density. Biophys J. 2003;84:3816–3828. doi:10.1016/S0006-3495(03)75109-1. PMC1302963 12770887

[22] Tuluc P, Kern G, Obermair GJ, et al Computer modeling of siRNA knockdown effects indicates an essential role of the Ca2+ channel alpha2delta-1 subunit in cardiac excitation-contraction coupling. Proc Natl Acad Sci USA. 2007;104:11091–11096. doi:10.1073/pnas.0700577104. PMC1904133 17563358

[23] Tang ZZ, Hong X, Wang J, et al Signature combinatorial splicing profiles of rat cardiac- and smooth-muscle Cav1.2 channels with distinct biophysical properties. Cell Calcium. 2007;41:417–428. doi:10.1016/j.ceca.2006.08.002. 16979758

[24] Tang ZZ, Liao P, Li G, et al Differential splicing patterns of L-type calcium channel Cav1.2 subunit in hearts of spontaneously hypertensive rats and wistar kyoto rats. Biochim Biophys Acta. 2008;1783:118–130. doi:10.1016/j.bbamcr.2007.11.003. 18070605

[25] Beyl S, Kügler P, Hohaus A, et al Methods for quantification of pore-voltage sensor interaction in Ca(V)1.2. Pflugers Arch. 2014 Feb;466(2):265–274. doi:10.1007/s00424-013-1319-8. PMC3902079 23873350

[26] Beyl S, Hohaus A, Andranovits S, et al Upward movement of IS4 and IIIS4 is a rate-limiting stage in Cav1.2 activation. Pflugers Arch. 2016;468:1895–1907. doi:10.1007/s00424-016-1895-5. PMC5138263 27796578

[27] Rogers JC, Qu Y, Tanada TN, et al Molecular determinants of high affinity binding of a-scorpion toxin and sea anemone toxin in the S3-S4 Extacellular loop in domain IV of the Na+ channel a subunit. J Biol Chem. 1996;271:15950–15962. doi:10.1074/jbc.271.27.15950. 8663157

[28] Koschak A, Reimer D, Huber I, et al Alpha 1D (Cav1.3) subunits can form l-type Ca2+ channels activating at negative voltages. J Biol Chem. 2001;276:22100–22106. doi:10.1074/jbc.M101469200. 11285265

[29] Grabner M, Dirksen RT, Beam KG Tagging with green fluorescent protein reveals a distinct subcellular distribution of L-type and non-L-type Ca2+ channels expressed in dysgenic myotubes. Proc Natl Acad Sci USA. 1998;95:1903–1908. doi:10.1073/pnas.95.4.1903. PMC19211 9465115

[30] Powell JA, Petherbridge L, Flucher BE Formation of triads without the dihydropyridine receptor subunits in cell lines from dysgenic skeletal muscle. J Cell Biol. 1996;134:375–387. doi:10.1083/jcb.134.2.375. PMC2120881 8707823

[31] Obermair GJ, Kugler G, Flucher BE The role of the calcium channel α2δ-1 subunit in skeletal muscle. J Muscle Res Cell Motil. 2004;25. PMID: 15467389. 10.1023/b:jure.0000038361.47060.fe 15467389

[32] Neuhuber B, Gerster U, Döring F, et al Association of calcium channel α1S and β1a subunits is required for the targeting of β1a but not of α1S into skeletal muscle triads. Proc Natl Acad Sci USA. 1998;95:5015–5020. doi:10.1073/pnas.95.9.5015. PMC20205 9560220

[33] Campiglio M, Flucher BE STAC3 stably interacts through its C1 domain with CaV1.1 in skeletal muscle triads. Sci Rep. 2017;7:41003. doi:10.1038/srep41003. PMC5253670 28112192

[34] Campiglio M, Di Biase V, Tuluc P, et al Stable incorporation versus dynamic exchange of β subunits in a native Ca2+ channel complex. J Cell Sci. 2013;126:2092–2101. doi:10.1242/jcs.jcs124537. PMC4148589 23447673

